# Management of Chemotherapy-Induced Left Ventricular Dysfunction and Heart Failure in Patients With Cancer While Undergoing Cancer Treatment: The MD Anderson Practice

**DOI:** 10.3389/fcvm.2018.00024

**Published:** 2018-03-28

**Authors:** Anecita P. Fadol

**Affiliations:** Department of Nursing, University of Texas MD Anderson Cancer Center, Houston, TX, United States

**Keywords:** chemotherapy-induced left ventricular dysfunction, heart failure, cancer, patient-centered care, interdisciplinary

## Abstract

Chemotherapy-induced cardiotoxicity resulting in heart failure (HF) is one of the most dreaded complications of cancer therapy that can significantly impact morbidity and mortality. With a high prevalence of cardiovascular disease in cancer patients, the risk of developing HF is significantly increased. A new discipline of Onco-Cardiology has evolved to address the cardiovascular needs of patients with cancer, however, there is limited evidence-based data to guide clinical decision-making in the management of the cardiovascular complications of cancer therapy. The department of cardiology at MD Anderson Cancer Center initiated the MD Anderson Practice (MAP) project and developed algorithms to guide the management of the cardiovascular complications of cancer therapy. For chemotherapy-induced HF, we initiated the Heart Success Program (HSP), a patient-centered program that promotes interdisciplinary collaboration for the management of concurrent HF resulting from chemotherapy-induced cardiotoxicity. After one year of HSP implementation, compliance with the Center for Medicare and Medicaid Services HF core measures has significantly improved. The measurement of LVEF and initiation of recommended pharmacologic therapy for HF (angiotensin converting enzyme inhibitor [ACE-I] or angiotensin receptor blocker for ACE-I intolerant patients) has improved to 100%; provision of discharge instruction has improved from 50 to 94%; and the 30-day hospital readmission rate decreased from 40 to 27%. This article will describe the MD Anderson Practice in the management of chemotherapy-induced cardiomyopathy and HF in cancer patients through the HSP. The novelty of the HSP has raised clinician’s awareness of the magnitude of the clinical problem of HF in cancer and the

## Introduction

**Figure 1:**
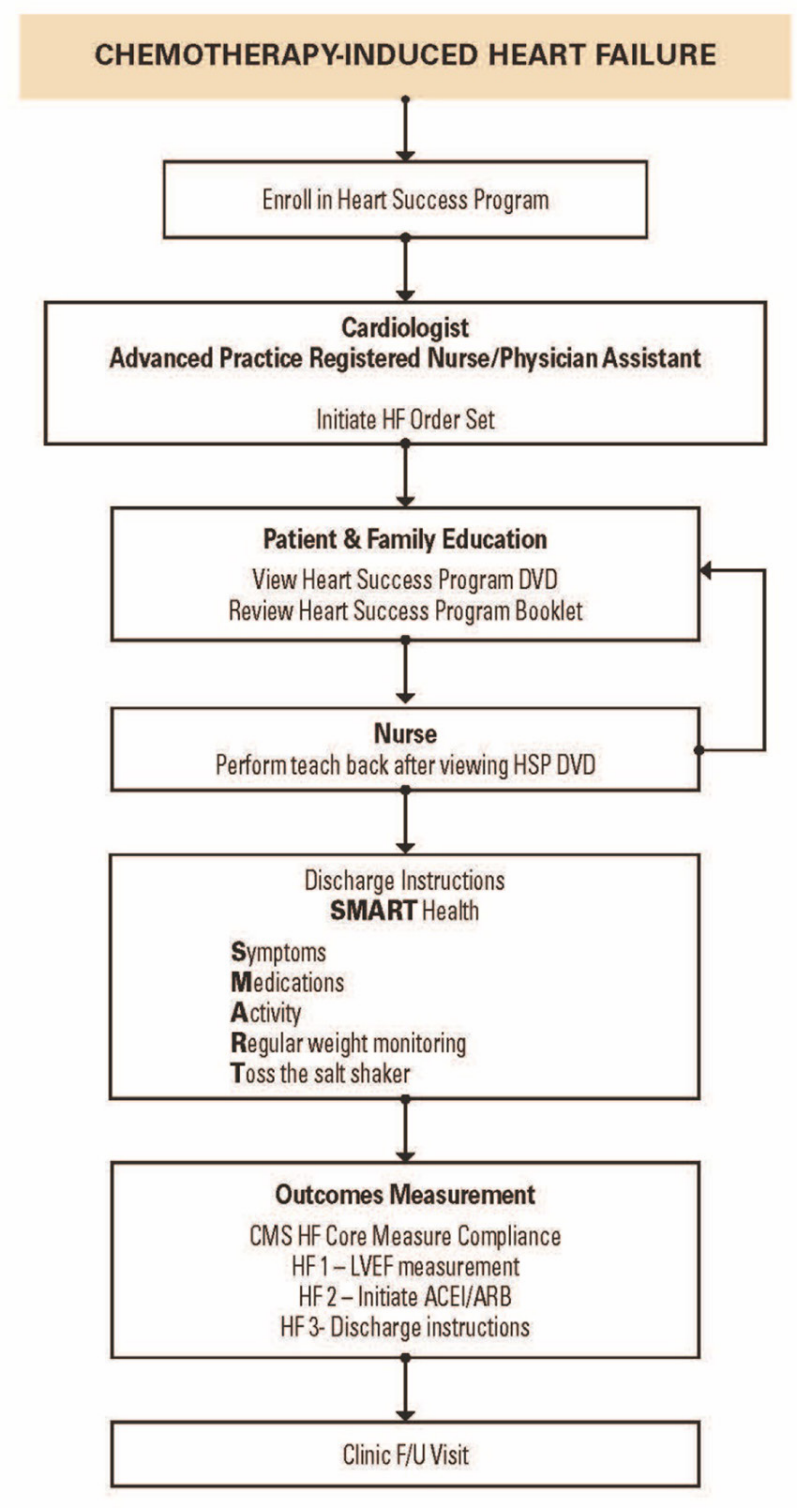
The MD Anderson Practice (MAP) for Management of Chemotherapy-Induced Heart Failure (10).

Cardiotoxicity resulting in heart failure (HF) is one of the most dreaded complications of cancer therapy that can significantly impact patient care and clinical outcomes. HF can occur as a consequence of cancer treatment–related cardiotoxicity secondary to chemotherapy (1, 2), radiation therapy (3) and biotherapy (4). A majority of cancer patients are usually older at the time of their cancer diagnosis and with multiple co-morbidities associated with aging which can increase the complexity of care. Additionally, cancer patients have a high prevalence of baseline cardiovascular risk factors and cardiovascular disease (CVD) prior to initiation of cancer therapy, making them more vulnerable to cardiovascular injuries, which may increase their risk of developing cardiomyopathy leading to HF and death. Management of patients with multiple comorbidities requires multiple providers and specialists which can predispose to the risk of fragmented and inefficient care (5). Over the course of cancer therapy, cancer patients with multiple comorbidities are managed by multidisciplinary teams with a concentrated effort on treating cancer, and the focus on the cardiotoxic effects of cancer treatments may be minimalized which can result in adverse cardiac outcomes and unplanned hospitalizations. A new discipline of Onco-Cardiology or Cardio-Oncology has evolved to address the cardiovascular needs of patients with cancer and optimize their care in a multidisciplinary approach. However, there is limited evidence-based data to guide clinical decision-making in many areas of Onco-Cardiology, although a number of documents attempting to define best practices from national societies is increasing (6–9).Because cancer patients are often excluded from cardiology trials, purely evidence-based data in the management of the cardiac complications of cancer therapy is almost impossible. At MD Anderson, we initiated the MD Anderson Practice (MAP) project to summarize our practice patterns into algorithms based on our cumulative experience from our everyday practice in the management of cancer patients. This article will describe the MD Anderson Practice (MAP) (Figure 1) in the management of chemotherapy-induced cardiomyopathy and HF in cancer patients through the Heart Success Program.

## The Heart Success Program

**Figure 2:**
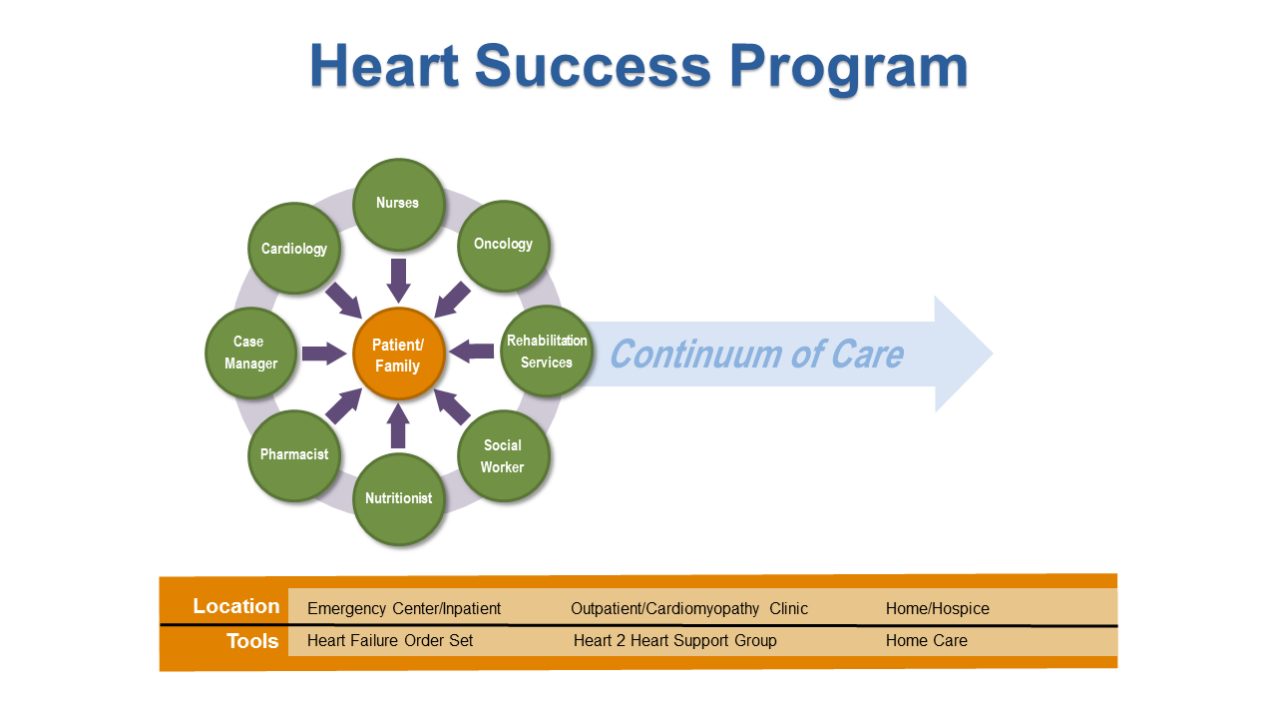
The Heart Success Program.

The high level of complexity in the care of patients with cancer and concurrent HF requires a comprehensive interdisciplinary approach to provide appropriate clinical management so that patients can continue to receive cancer treatment to improve survival and patient’s quality of life. To promote collaboration among cardiologists, oncologists, nurses and other members of the health care team, we developed the Heart Success Program (HSP) (Figure 2), a patient-centered, interdisciplinary program to coordinate the management of concurrent cardiomyopathy (CMP) and HF while the patient is receiving cancer treatment. The HSP is based on the principles of disease management to bridge the gap between existing therapeutic options for cardiotoxicity and the realities of clinical practice of the new specialty of Onco-Cardiology. Evidence from meta-analyses of disease management programs have shown that such interventions has a significant impact on survival and hospitalization rates (11–15). The goals of the HSP are to: (1) identify cancer patients whose clinical history and oncologic treatment put them at higher risk for developing cardiomyopathy and HF while receiving cancer treatment; (2) provide timely initiation of recommended pharmacologic therapy for patients who developed chemotherapy-induced HF based on available research data; and (3) actively involve patients in the management of their illness through comprehensive patient education. This interdisciplinary approach fosters open communication and collaborative decision- making to develop the best plan of care for the patient. The HSP exemplifies the Institute of Medicine (IOM) recommendation of “Delivering High-Quality Cancer Care,” to address the complex care needs of persons with multiple coexisting diseases, increased side effects from treatment, and greater need for social support (16).

### Identification and Monitoring of Patients at Risk for Chemotherapy-Induced cardiomyopathy/Heart Failure

According to the American College of Cardiology and American Heart Association Heart Failure guidelines (17), patients receiving chemotherapy may be considered a stage A heart failure with an increased risk of developing left ventricular dysfunction (LVD). Identification of cancer patients at high risk for chemotherapy-induced cardiotoxicity is one key strategy to reduce morbidity and mortality from cardiovascular toxicity related to cancer therapy. A pre-existing cardiovascular risk factor is itself a strong predictor for the development of cardiovascular injury after chemotherapy, making the likely risk for cardiovascular disease much greater. However, clinical guidelines for screening and monitoring of cardiotoxicity during and after cancer therapies in adults are lacking. Certain professional organizations such as the European Society of Cardiology (ESC) (8) and the American Society of Clinical Oncology (ASCO) (18) have suggested recommendations for prevention and monitoring of cardiac dysfunction in patients with cancer and survivors of adult-onset cancers.

In the HSP, the interdisciplinary team are actively involved with screening patients for cardiac risk factors before receiving cancer treatments, particularly chemotherapeutic agents with potential cardiotoxicity. Patients with cardiac risk factors (e.g., diabetes mellitus, dyslipidemia, hypertension, smoking history, and obesity [BMI >30], etc.), or with history of cardiovascular disease (coronary artery disease (CAD), valvular disease, myocardial infarction, cardiomyopathy) are more vulnerable to cardiovascular injuries and increase their risk of premature cardiovascular death. During the oncologic-active treatment phase, every effort is made to continue and optimize the therapy of underlying cardiovascular diseases, as well as to correct pre-existing and newly acquired cardiovascular risk factors.

The most classic and frequent clinical manifestation of cardiotoxicity is the development of left ventricular dysfunction (LVD). All high risk patients are screened for LVD with an echocardiography with global longitudinal strain measurement. The development of LVD, particularly systolic dysfunction (LVEF <50%) even when asymptomatic, not only negatively affects patients’ cardiologic outcome, but it also seriously limits their therapeutic opportunities when adjunctive chemotherapy is required. Moreover, the presence of impaired cardiac function restricts the choice of possible oncologic treatments to those considered less aggressive and, consequently, less effective. If the LVEF is ≤50%, the oncologist formulate the cancer therapeutic options in collaboration with the cardiologist, taking into consideration the patient’s cardiac function. Nurses routinely screen patients and ensure that the Onco –Cardiology team are informed of patients who developed chemotherapy-induced CMP/HF, and patients with co-morbidities or CVD risk factors that are not optimally controlled.

### Timely Initiation of Pharmacologic Therapy for Heart Failure

Interventions in HF management have become increasingly complicated for patients, in part due to multiple-drug therapy and complex regimens. Guidelines from both European and US cardiology societies do not provide specific recommendations for cancer patients who develop HF after cancer treatment (19, 20). However, small studies have shown that patients with decreased LVEF (≤50%) who are started on standard HF pharmacotherapy, including angiotensin-converting enzyme (ACE) inhibitors and beta blockers have shown improvement in cardiac function (21–24). A prospective study on cancer patients with anthracycline-induced cardiomyopathy demonstrated that the time elapsed from the end of chemotherapy to the start of HF therapy with ACE inhibitors and, when tolerated, with beta-blockers, was a crucial variable for recovery of cardiac dysfunction (23, 25). This finding emphasizes the crucial importance of the early detection of cardiotoxicity and timely initiation of HF pharmacotherapy, and therefore should always be considered and attempted in all cases of anthracycline –induced cardiomyopathy. The clinical pharmacist work collaboratively with other members of the healthcare team particularly with the initiation and titration of drug regimens based on specific patient characteristics. In addition, the clinical pharmacist is accountable for determining the presence of specific drug-related problems (e.g., drug and/or allergy interactions, medical conditions without drug therapy, drugs without indications, therapeutic duplication, the presence or potential for drug-drug, drug-lab, or drug-food interactions, and the presence or potential for adverse reactions) in all HF patients. Moreover, the clinical pharmacist provides education to other healthcare professionals.

### Patient/Family Education

After the patient is confirmed with a HF diagnosis, the inpatient HF order set is activated. The nurses initiate patient and family education and encourage patients to view a 15 min video presentation entitled, “Heart Success for Cancer Patients” (26). This video was developed at MD Anderson specifically for patients with cancer and HF. The video shows important information regarding HF, chemotherapeutic agents with potential cardiotoxicity, HF medications and adverse effects, signs and symptoms of HF exacerbation, and when to seek medical care. Individualized patient and family education are essential components of the Heart Success Program that enables patients to become active “co-managers” of their disease. Patient teaching is reinforced with the “Teach-Back” method, also referred to as “closing the loop”, which allows patients to articulate, in their own words, their understanding of what they were taught by providers (27). The “teach back” process enables nurses to identify areas of HF management that patients are deficient and need further explanation. A copy of the patient education booklet “Heart Success: A Resource Guide for Individuals Living with Cancer and Heart Failure” (28) is provided to the patient and family when HF education is initiated. The patient education booklet highlights the essential learning points in the videotape and can be used by patients for reference at home after hospital discharge. Empowering patients requires increasing their comprehension of the disease process and managing their common symptoms such as decreasing salt intake to prevent lower extremity edema and shortness of breath, which can prevent unplanned hospital readmission.

Dietary issues of patients with cancer and HF is a major challenge. The oncology patient often have gastrointestinal symptoms related to cancer and adverse effects of cancer treatment which can have a major impact on the patient’s nutritional status. The nutritionist has a major role in assisting patients with food choices to meet their nutritional needs, without compromising taste particularly with low sodium diet for patients with HF.

Prior to discharge, the nurses review with patients the essential information to guide them with their care at home using the “Heart SMART” guide. SMART is an acronym developed to help patients easily recall essential information related to symptoms, medications, activity, regular weight monitoring, and toss the salt shaker (for low sodium diet). Patients are also provided with instructions on what to do if symptoms worsen (20). Comprehensive discharge planning and post-discharge support for patients with heart failure has been shown to significantly reduce hospital readmission rates (12).

Successful transition to home after hospital discharge is critical to prevent unplanned hospital readmission. The case manager and social worker work collaboratively to facilitate the process, which includes assessment, planning and facilitating options and services to meet the individual’s cardiac and oncology needs. Timely planning and intervention regarding available resources prevents unnecessary confusion and decreases stress for patients and families. The case manager also acts as a link between the hospital and the agencies when referrals are initiated. Participation in weekly HSP rounds allows discussions between the multidisciplinary team regarding interventions, and therefore promotes continuity of care.

### Outcomes Measurement

Although specialty hospitals such as MD Anderson are currently exempt from public reporting of HF core measures as required by the Center for Medicare and Medicaid Services (CMS), we monitored the core measures with the HSP implementation which includes: (1) measurement of left ventricular ejection fraction (LVEF); (2) initiation of ACE-I or an angiotensin-receptor blocker for patients with LVD; and (3) discharge instructions. After one year of implementing the HSP in a medical telemetry unit, we reviewed the database to evaluate our compliance with the CMS core measures for HF. Our compliance with measurement of LVEF and initiation of ACE-I/ARB was 100%. The provision of discharge instruction has improved from 50 to 94% since the implementation of the HSP. The 30 day hospital readmission rate has also decreased from 40 to 27% since the implementation of the HSP in 2012. Using continuous improvement methodology, the team refined the tools based on the experience and lessons learned and disseminate the HSP to the other areas of the hospital, one clinical area at a time. Currently, the HSP is disseminated throughout MD Anderson. The HSP is a quality improvement (QI) initiative developed to promote higher standards in the management of cancer patients.

## Conclusion

The growing awareness about cardiovascular side effects of anticancer drugs, and the increasing number of cancer survivors entails a host of novel challenges. Cardiovascular safety represents an emerging problem for patients with cancer and cancer survivors. The prevalence of cancer treatment–related cardiovascular disease is increasing, and its management demands a multidisciplinary approach from cardiologists, oncologists and the interdisciplinary team involved in the management of these patients. It is no longer sufficient to focus exclusively on the cancer diagnosis and associated cancer treatments. Providers must enlarge their focus to include pre-existing chronic illnesses as well as cancer treatment–related illness and disability. The novelty of the Heart Success Program has raised clinician’s awareness of the magnitude of the clinical problem of heart failure in cancer and the importance of interdisciplinary collaboration to improve clinical outcomes. The HSP provides a model for engaging patients and family members as partners with a shared goal of reducing the burden of HF in people with cancer.

## Author Contributions

AF is responsible for the conception of the project, drafting, revising and final approval of the manuscript prior to submission.

## Conflict of Interest Statement

The author declares that the research was conducted in the absence of any commercial or financial relationships that could be construed as a potential conflict of interest.
